# Renadirsen, a Novel 2′OMeRNA/ENA^®^ Chimera Antisense Oligonucleotide, Induces Robust Exon 45 Skipping for Dystrophin In Vivo

**DOI:** 10.3390/cimb43030090

**Published:** 2021-09-25

**Authors:** Kentaro Ito, Hideo Takakusa, Masayo Kakuta, Akira Kanda, Nana Takagi, Hiroyuki Nagase, Nobuaki Watanabe, Daigo Asano, Ryoya Goda, Takeshi Masuda, Akifumi Nakamura, Yoshiyuki Onishi, Toshio Onoda, Makoto Koizumi, Yasuhiro Takeshima, Masafumi Matsuo, Kiyosumi Takaishi

**Affiliations:** 1 Specialty Medicine Research Laboratories I, Daiichi Sankyo Co., Ltd., Shinagawa, Tokyo 1408710, Japan; ito.kentaro.pb@daiichisankyo.co.jp (K.I.); kanda.akira.xw@daiichisankyo.co.jp (A.K.); nagase.hiroyuki.z8@daiichisankyo.co.jp (H.N.); 2 Drug Metabolism and Pharmacokinetics Research Laboratories, Daiichi Sankyo Co., Ltd., Shinagawa, Tokyo 1408710, Japan; takakusa.hideo.yb@daiichisankyo.co.jp (H.T.); watanabe.nobuaki.nc@daiichisankyo.co.jp (N.W.); asano.daigo.vi@daiichisankyo.co.jp (D.A.); goda.ryoya.u4@daiichisankyo.co.jp (R.G.); 3 Medical Information Department, Daiichi Sankyo Co., Ltd., Chuo, Tokyo 1038426, Japan; kakuta.masayo.c2@daiichisankyo.co.jp; 4 Safety and Risk Management Department, Daiichi Sankyo Co., Ltd., Chuo, Tokyo 1038426, Japan; takagi.nana.x6@daiichisankyo.co.jp; 5 Modality Research Laboratories, Daiichi Sankyo Co., Ltd., Shinagawa, Tokyo 1409710, Japan; masuda.takeshi.f8@daiichisankyo.co.jp (T.M.); nakamura.akifumi.ay@daiichisankyo.co.jp (A.N.); onishi.yoshiyuki.a2@daiichisankyo.co.jp (Y.O.); koizumi.makoto.h7@daiichisankyo.co.jp (M.K.); 6 Intellectual Property Department, Daiichi Sankyo Co., Ltd., Shinagawa, Tokyo 1409710, Japan; onoda.toshio.zi@daiichisankyo.co.jp; 7 Department of Pediatrics, Hyogo College of Medicine, Nishinomiya 6638501, Japan; ytake@hyo-med.ac.jp; 8 Research Center for Locomotion Biology, Kobe Gakuin University, Nishi, Kobe 6512180, Japan; matsuo@kobe-u.ac.jp

**Keywords:** Duchenne muscular dystrophy, exon skipping, antisense oligonucleotide, dystrophin, 2′-*O*,4′-*C*-ethylene-bridged nucleic acid (ENA), renadirsen sodium, cardiac muscle

## Abstract

Duchenne muscular dystrophy (DMD) is a progressive muscle-wasting disease caused by out-of-frame or nonsense mutation in the dystrophin gene. It begins with a loss of ambulation between 9 and 14 years of age, followed by various other symptoms including cardiac dysfunction. Exon skipping of patients’ *DMD* pre-mRNA induced by antisense oligonucleotides (AOs) is expected to produce shorter but partly functional dystrophin proteins, such as those possessed by patients with the less severe Becker muscular dystrophy. We are working on developing modified nucleotides, such as 2′-*O*,4′-*C*-ethylene-bridged nucleic acids (ENAs), possessing high nuclease resistance and high affinity for complementary RNA strands. Here, we demonstrate the preclinical characteristics (exon-skipping activity in vivo, stability in blood, pharmacokinetics, and tissue distribution) of renadirsen, a novel AO modified with 2′-*O*-methyl RNA/ENA chimera phosphorothioate designed for dystrophin exon 45 skipping and currently under clinical trials. Notably, systemic delivery of renadirsen sodium promoted dystrophin exon skipping in cardiac muscle, skeletal muscle, and diaphragm, compared with AOs with the same sequence as renadirsen but conventionally modified by PMO and 2′OMePS. These findings suggest the promise of renadirsen sodium as a therapeutic agent that improves not only skeletal muscle symptoms but also other symptoms in DMD patients, such as cardiac dysfunction.

## 1. Introduction

Duchenne muscular dystrophy (DMD) (OMIM 310200) is the most common fatal muscular disease in children, affecting more than one in every 3500 boys [1,2]. DMD patients show progressive muscle wasting and generally experience difficulty walking, declines in respiratory and cardiac functions, and premature death with a life expectancy of about 30 years [3,4]. DMD is caused by frameshift or nonsense mutation in the dystrophin gene, which leads to truncated, nonfunctional dystrophin proteins [5]. Some antisense oligonucleotides (AOs) have been found to convert out-of-frame deletion into in-frame deletion by inducing skipping of the exon adjacent to the deletion domain, which produces truncated but still functional dystrophin proteins [6,7,8]. To treat DMD, negatively charged 2′-*O*-methyl RNA oligonucleotides with all phosphorothioate bonds (2′OMePSs) and neutral phosphorodiamidate morpholino oligomers (PMOs) have been developed as advanced exon-skipping AOs in order to enhance the binding ability and stability [9,10,11]. However, recent studies have failed to show marked clinical benefit, probably due to insufficient restoration of dystrophin expression. Drisapersen, a 2′OMePS targeting exon 51 of dystrophin, failed to obtain approval from the US Food and Drug Administration (FDA) after clinical studies showed no clear benefit in terms of specific clinical endpoints, such as changes in a 6-min walk test, following 24-week treatment [12]. Although PMO-backbone AOs such as eteplirsen (EXONDYS 51), golodirsen (Vyondys 53), viltolarsen (Viltepso), and casimersen (Amondys 45) have been conditionally approved by the FDA, further follow-up trials have been requested to assess their effects on motor function [10,12]. Furthermore, preclinical studies using mice have indicated that both 2′OMePS and PMO therapeutics are barely effective in these animals, especially for treating cardiac muscle damage at clinically feasible doses [13,14,15,16]. This is important as the early death of patients with DMD is caused by wasting of the respiratory or cardiac muscles. In the past decade, the life expectancy of patients with DMD has been increased from 15 to 19 to >30 years by general medical treatments and physical therapy, and cardiac involvement has been demonstrated as a major cause of morbidity and mortality in such patients.

To overcome the above-mentioned issues with AOs, several modified nucleic acids have been created to provide higher nuclease resistance and high affinity for complementary sequences. 2′-*O*,4′-*C*-ethylene-bridged nucleic acid (ENA^®^; a registered trademark of Daiichi Sankyo Co., Ltd., Tokyo, Japan) has been developed to confer higher nuclease resistance and higher affinity for complementary RNA strands than the AOs modified with 2′OMePSs or PMOs [17,18,19,20,21]. Previously, we reported on AO85, an 18-mer AO with 2′OMeRNA/ENA on the phosphodiester backbone, targeting sequences conserved in both humans and mice in exon 45 of dystrophin. AO85 showed high dystrophin restoration in several DMD-patient-derived myocytes and had high tolerability in a repeated-administration toxicity study in mice [22]. In one clinical study testing the distance walked in 6 min, AO85 was reported to increase this distance and to be well tolerated when injected intravenously at a dose of 0.5 mg/kg/week [23]. Renadirsen sodium (alternative code names: DS-5141b and AO88) is a novel 18-mer AO with 2′OMeRNA/ENA on the phosphorothioate backbone, which has the same sequence as AO85 [24]. It is currently under clinical trials in Japan (ClinicalTrials.gov: NCT02667483). In preclinical studies, renadirsen sodium has demonstrated substantial dystrophin restoration via exon 45 skipping, suppression of Ca^2+^ overflow, and secretion of creatine kinase (CK) in myotube cells derived from DMD patients iPS cells [25].

In the present study, we reveal the characteristics of renadirsen sodium in in vivo studies. Further, subcutaneous administration of renadirsen sodium in *mdx* mice, the most widely used animal model in DMD research [26], here demonstrated robust exon-skipping activity in various target muscles, including the heart, in comparison to conventionally modified AOs (2′OMePS and PMO) possessing the same sequence as renadirsen. These findings indicate that renadirsen sodium may provide substantial benefits for treating muscle wasting, including cardiomyopathy, in patients with DMD.

## 2. Materials and Methods

### 2.1. Oligonucleotide Synthesis

Renadirsen and 2′OMePS-e45 were synthesized using phosphoramidite methods as follows [19,20]. A 2-hydroxyethyl phosphate group at the 3′-end of renadirsen was synthesized using the corresponding phosphoramidite reagent, as described elsewhere [27]. PMO-e45 was synthesized in accordance with a previously reported procedure [28]. [^14^C]Renadirsen was synthesized using protected [2-^14^C]2′-*O*-methyluridine phosphoramidite, which was incorporated into position 3 from the 3′-end of the nucleotide sequences.

### 2.2. Drug Administration to Mice for RNA Extraction

Male C57BL/6NCrCrlj mice (wild type, WT) and dystrophin-deficient *mdx* mice (C57BL/10ScSn-Dmd<mdx>/J) were purchased from Charles River Laboratories Japan, Inc., and the Jackson Laboratory, respectively. The animals were quarantined and acclimated with feeding for 7 days. All animals were provided access to standard chow and water ad libitum. WT or *mdx* mice at 5 weeks of age were injected subcutaneously with 1–30 mg/kg renadirsen sodium, 2′OMePS-e45, or PMO-e45 dissolved in sterile saline once or once a week for 4 weeks. Sham-injected controls received saline alone at 10 mL/kg. Injection was performed subcutaneously into the back of each mouse. The mice were euthanized 1 week after the final administration, and tissues were harvested and stored at −80 °C until further analysis.

### 2.3. RNA Extraction and RT-PCR

The tissues were sliced into sections less than 5 mm thick, placed into a tube containing TRIzol reagent (Thermo Fisher Scientific, Waltham, MA, USA), and homogenized using a Shake Master Neo (BMS-M10N21; Biomedical Science, Tokyo, Japan). Total RNA extraction was performed using a PureLink RNA Mini kit (Thermo Fisher Scientific), PureLink DNase (Thermo Fisher Scientific), RNeasy Mini kit (QIAGEN, Valencia, CA, USA), and RNeasy-Free DNase Set (QIAGEN), as per the manufacturer’s recommendations. The prepared total RNA was quantified using a spectrophotometer (NanoDrop Lite; Thermo Fisher Scientific). The total RNA solution was stored at −80 °C until further analysis.

One-step RT-PCR was carried out with the OneStep RT-PCR Kit (QIAGEN) in a 25 µL reaction mixture containing 200 ng of total RNA. RT-PCR to detect skipping of exon 45 in mouse was performed using a thermal cycler (2720; Thermo Fisher Scientific) with the following thermal profile: 1 cycle of 50 °C for 30 min and 95 °C for 15 min; 40 cycles of 94 °C for 30 s, 58 °C for 30 s, and 72 °C for 1 min; and then 10 min at 72 °C using a forward primer corresponding to part of the sequence in exon 44 (GTTGAACAGTTTTTCAAAAAGACACAA) and a reverse primer corresponding to part of the sequence in exon 47 (TCCACTGGAGATTTGTCTGT). The sizes of the amplicons from the intact and skipped sequences were estimated based on the sequence registered in the GenBank database (NM_007868) with each primer sequence.

The RT-PCR products were analyzed using an Agilent 2100 Bioanalyzer with the Agilent DNA1000 kit (Agilent Technologies, Santa Clara, CA, USA). This system produced gel images of the RT-PCR product and determined the concentration of the product corresponding to each band in these images. The total amount of PCR product corresponding to exon-45-skipped dystrophin was calculated by multiplying the obtained concentration of this product by the volume of the PCR reaction solution. For normalization, the concentration of amplicon with no peak detected was treated as 0. If no peak was detected for all individuals in the group, the mean for that group was displayed as “not detected” or “N.D.”.

### 2.4. In Vitro Stability Assay in Mouse Blood

Mouse blood was obtained from male WT mice (*n* = 42) under isoflurane anesthesia. An aliquot of renadirsen sodium solution was added to the freshly obtained pooled mouse blood at a final concentration of 2.0 μg/mL, which was divided into three portions prior to incubation. Aliquots of the blood incubation mixture were taken 0, 1, 2, 4, and 8 h after incubation at 37 °C. An aliquot (100 μL) of each blood sample was mixed with 100 μL of aqueous methanol containing an internal standard (IS) compound, followed by filtration and concentration using Amicon Ultra filters (YM-50 and YM-3, respectively). The samples were analyzed using an LC-MS/MS system (HPLC: Ultimate, MS: TSQ Vantage; Thermo Fisher Scientific), and the peak area ratio (renadirsen/internal standard (IS)) was determined. The data at each time point are expressed as stability %, with the peak area ratio at 0 min being set as 100%.

### 2.5. Pharmacokinetics of Renadirsen in Mice

Renadirsen sodium was dissolved in saline at a concentration of 10 mg/mL and then serially diluted with physiological saline solution to produce solutions with concentrations of 3 and 1 mg/mL for dosing. These solutions were administered subcutaneously at 10 mL/kg into the back of each male *mdx* mice at 5 weeks of age. Blood samples were collected 5, 15, and 30 min, and 1, 2, 4, 8, and 24 h after administration and centrifuged (13,200× *g*, 6 min, 4 °C) to obtain plasma. The plasma samples were pretreated with a Clarity OTX 100 mg/3 mL cartridge (Phenomenex Inc., Torrance, CA, USA) and then analyzed using an LC-MS/MS system [LC: NANOSPACE SI-2 system (Shiseido Co., Ltd., Tokyo, Japan), MS: Triple Quad 5500 (AB Sciex)]. Chromatographic separation was performed using a Proteonavi column (2.0 mm I.D. × 50 mm, particle size 5 μm; Shiseido Co., Ltd., Tokyo, Japan) and ion-pair eluents (1,1,1,3,3,3-hexafluoro-2-propanol and triethylamine). Peaks on the MRM chromatogram, which were detected using the MRM mode, were identified based on the eluting position and the monitoring ions. The concentration of analyte was determined from the ratio of the peak area of analyte to that of the IS using the internal standard method. The equations for the calibration curves, concentrations of quality control (QC) samples, and pharmacokinetics (PK) samples were calculated using Analyst software (version 1.5.2; AB SCIEX). The equation for the calibration curve was obtained from the relationship between the ratio of the analyte peak area to the IS peak area and nominal concentrations of the analyte, using the least squares method (with 1/×^2^ weight).

### 2.6. In Vitro Protein Binding of ^14^C-Labeled Renadirsen Sodium to Mouse Plasma

[^14^C]Renadirsen sodium (7.2 μCi/mg) was weighed and dissolved in distilled water to prepare a 10 mg/mL solution, and was serially diluted with distilled water to make solutions with concentrations of 1000 and 100 μg/mL. After fasting for approximately 17 h, blood was collected from the inferior vena cava of each male mouse [Crlj: CD1(ICR), male] under isoflurane inhalation using a syringe with an injection needle treated with heparin lithium. The blood was injected into a vacuum tube containing heparin lithium and centrifuged (1800× *g*, 4 °C, 15 min) to separate plasma. The test plasma sample prepared at each [^14^C]renadirsen sodium concentration was treated as an initial sample. Each of the test plasma samples was injected into an ultracentrifugation tube (0.23PA tube; Hitachi Koki, Tokyo, Japan). Aliquots of the test plasma samples were transferred into four ultracentrifugation tubes for the 100 and 10 μg/mL samples and eight ultracentrifugation tubes for the 1 μg/mL samples. The samples in the ultracentrifugation tubes were centrifuged (200,000× *g*, 4 °C, 16 h) to separate the supernatant. The supernatant was then collected as follows: the micropipette tip was held against the upper tube wall and the micropipette was placed immediately below the supernatant along the tube wall so that the lipid layer (uppermost part) was not drawn into the micropipette. When the micropipette was inserted into the supernatant and the supernatant was drawn up, it was confirmed that the protein layer (lower layer) and the lipid layer (upper layer) were not turbulent. The test plasma sample and recovered supernatant were dissolved in 2 mL of the tissue solubilizer Soluene-350 and mixed with Hionic-Fluor. The radioactivity (dpm) of each sample was measured with a liquid scintillation counter (1900CA, 2500TR, and 3100TR; PerkinElmer, Inc., Waltham, MA, USA) for 2 min. The external standard source method was used to correct for counting efficiency. The plasma protein binding ratio (%) was calculated from the concentration of radioactivity (dpm/mL) in the supernatant obtained after ultracentrifugation of the [^14^C]renadirsen-sodium-spiked plasma sample and that in the [^14^C]renadirsen-sodium-spiked plasma sample without ultracentrifugation.

### 2.7. Quantitative Whole-Body Autoradiography after Administration of ^14^C-Labeled Renadirsen Sodium to Mice

[^14^C]Renadirsen sodium was dissolved in physiological saline (Japanese Pharmacopoeia, Otsuka Pharmaceutical Factory, Inc., Tokyo, Japan) to achieve a concentration of 1 mg/0.266 MBq/mL. The dosing formulation was administered once subcutaneously into the back of each male C57BL/6 mouse at a dose of 10 mg/kg at 7 weeks of age. At the prescribed time points (0.5, 96, 168, and 336 h after administration), each animal was euthanized via the inhalation of carbon dioxide and frozen in a dry ice–acetone bath. The frozen carcass was embedded in ~4 w/v% CMC–Na solution to prepare a frozen block. In each block, the calibration blood samples of four different concentrations (two samples per concentration, eight samples in total) were embedded at two positions diagonally so that they could serve as coordinate lines to identify the tissue locations on the resulting radioluminogram. Thin sections (15 or 18 slices per animal, 30 μm thick) were prepared with a large-scale sliding cryomicrotome (CM3600; Leica Microsystems AG) and collected on adhesive tapes (Tesa4129; Tesa Tape K.K.). Each of the whole-body frozen sections was freeze-dried, covered with Diafoil film (4 μm thick; Mitsubishi Plastics, Inc., Tokyo, Japan), and placed on an imaging plate (IP, BAS-MS2040, 20 cm× 40 cm; Fujifilm Corporation, Tokyo, Japan). After 72 h of exposure in a lead-shielded box (BAS-SHB2040; Fujifilm Corporation), the radioluminogram on the imaging plate was scanned [gradation: 65,536 (16 bit), resolution: 50 μm, latitude: 4, sensitivity: 10,000] with a bio-imaging analyzer (BAS-2500; Fujifilm Corporation) to obtain a radioluminogram. The radioluminograms were obtained for five thin sections per animal. Each radioluminogram was analyzed with a QWBA image-analyzing system (SeeScan Ver. 2.1.2.19; LabLogic Systems Limited, Sheffield, UK) to determine the concentration of radioactivity per unit area [(PSL-BG)/mm^2^] of blood and tissues after correction with the background PSL (photostimulated luminescence) level, as determined on the same radioluminogram in the absence of the calibration blood or tissue samples. Calibration curves were constructed by linear regression analysis using four radioactivity concentrations (weighting: 1/Y) in the calibration blood sample [(PSL-BG)/mm^2^)] against those equivalent to renadirsen sodium in blood samples for calibration (ng eq./g). The correlation coefficient (r) of the calibration curve was confirmed to be 0.99 or higher. Furthermore, the back-calculated radioactivity concentration (ng eq./g) in each calibration blood sample of all IPs (thin sections) was calculated, and the relative error (R.E.) value for each mean value and the C.V. value among all IPs in each calibration blood sample were confirmed to be within 20% (within 30% at the minimum concentration of calibration blood samples). The concentration of radioactivity in tissue (ng eq./g) was calculated from the concentration of radioactivity per unit area [(PSL-BG)/mm^2^] of the tissue and the calibration curve obtained from the same radioluminogram. The analyzed tissues were identified locally or confirmed visually on the radioluminograms. The concentrations of radioactivity in tissues were calculated as renadirsen sodium equivalent concentrations (ng eq./g).

## 3. Results

### 3.1. Exon Skipping in Tibialis Anterior Muscle, Diaphragm, and Cardiac Muscle in WT and Mdx Mice after Single Administration of Renadirsen Sodium

**Figure 1:**
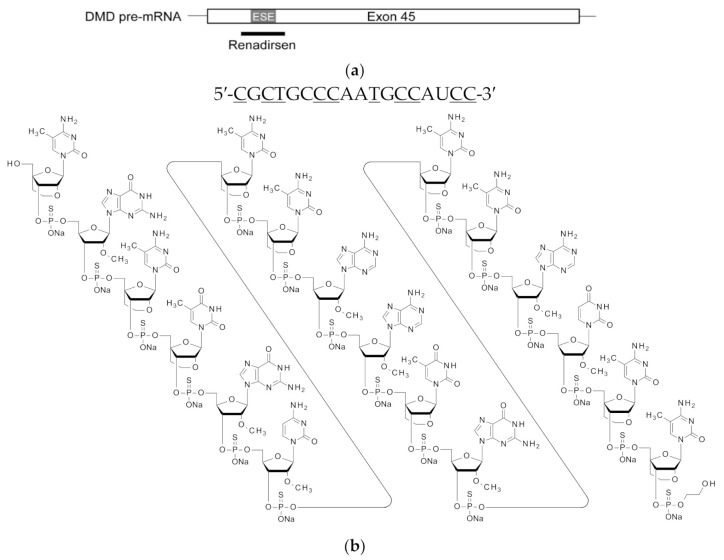
Schematic of the location, nucleotide sequence, and structure of renadirsen. (**a**) The location of renadirsen is schematically presented. Box and bars indicate exon 45 and flanking introns, respectively. The thick bar represents renadirsen. (**b**) Nucleotide sequence (upper) and structure (bottom) of renadirsen. Capital letters and underlined capital letters indicate 2′OMeRNA and ENA, respectively. ENA-C and ENA-T represent 2′-*O*,4′-*C*-ethylene-5-methylcytidine and 2′-*O*,4′-*C*-ethylene-5-methyluridine, respectively. A 2-hydroxyethyl phosphate group was attached at the 3′-end of renadirsen. All internucleotide bonds of renadirsen are phosphorothioate bonds.

**Figure 2:**
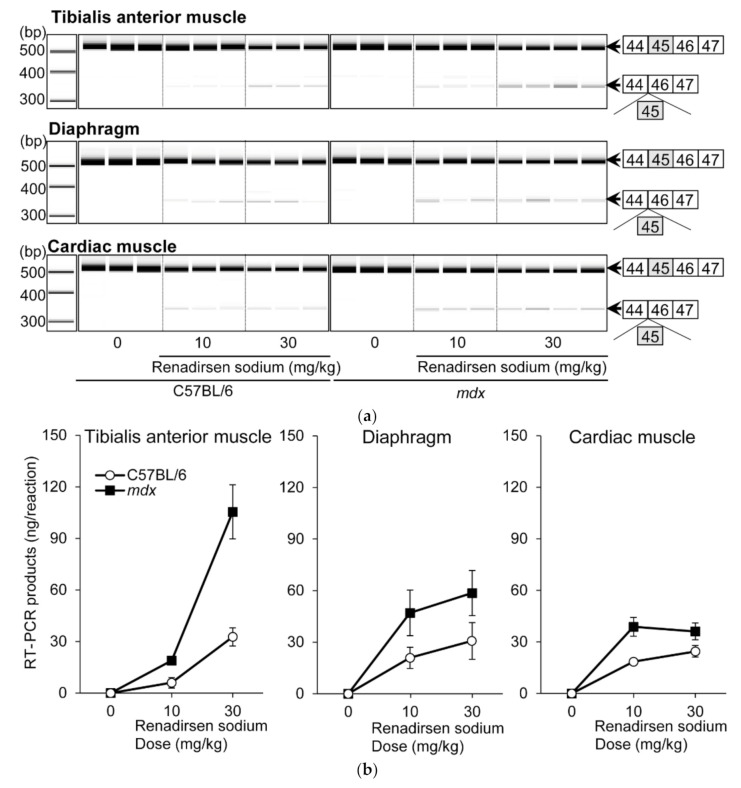
Exon 45 skipping in C57BL/6 and *mdx* mice after single s.c. administration of renadirsen sodium. (**a**) Detection of exon-45-skipped RT-PCR products of dystrophin mRNA in C57BL/6 and *mdx* mouse muscles after single s.c. administration. Total RNA was isolated from tibialis anterior muscle, diaphragm, and cardiac muscle. The structure of each PCR product is schematically shown at the right of the panel. (**b**) The concentrations of exon-45-skipped RT-PCR products of dystrophin mRNA from tibialis anterior muscle, diaphragm, and cardiac muscle were calculated using an Agilent 2100 Bioanalyzer. Data in (B) are shown as means ± SEM; *n* = 3–4.

Renadirsen sodium was screened as an exon-45-skipping AO that covers the splicing enhancer sequence of exon 45 [exonic splicing enhancer (ESE)], which is conserved in humans and mice (Figure 1a) [22]. Renadirsen sodium is an 18-mer AO designed as a 2′OMeRNA/ENA chimera in which all internucleotide bonds are phosphorothioate bonds. Eight cytosines and two uridines in renadirsen sodium were modified with ENA-C and ENA-T residues, respectively (Figure 1b). To evaluate the in vivo exon-skipping activity of renadirsen sodium in muscles, we administered it subcutaneously a single time at doses of 0, 10, and 30 mg/kg in male C57BL/6 and *mdx* mice. RT-PCR of total RNA extracted from the muscles 1 week after administration demonstrated that renadirsen sodium promoted exon 45 skipping of mouse dystrophin in all samples taken from the tibialis anterior muscle, diaphragm, and cardiac muscle in both C57BL/6 and *mdx* mice, even at a dose of 10 mg/kg (Figure 2a,b). Considering that PMO-based AOs generally do not induce exon-skipping activity in normal muscles and that little or no activity was observed in cardiac muscle following the administration of PMO- and 2′OMePSs-based AOs [15,16], it is notable that renadirsen sodium showed exon skipping in healthy muscles, including cardiac muscle, at a very low dose (10 mg/kg) with single systemic administration.

### 3.2. Stability and Pharmacokinetics of Renadirsen Sodium in Mice

**Figure 3:**
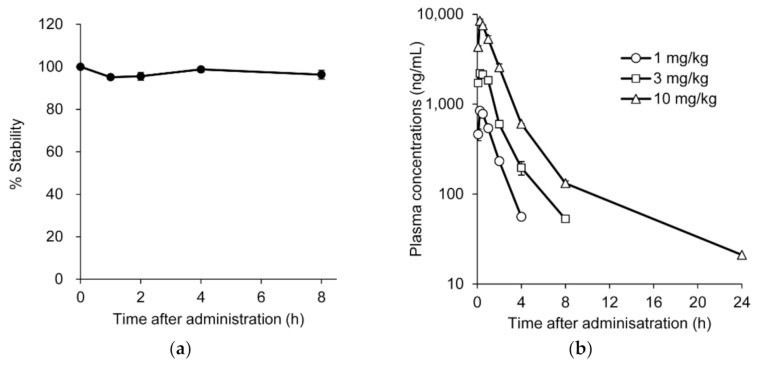
Stability and pharmacokinetics of renadirsen in mouse blood. (**a**) Stability of renadirsen in mouse blood was analyzed using an LC-MS/MS system, and peak area ratio (renadirsen/IS) was determined. The data at each time point are expressed as stability %, with peak area ratio at 0 min being 100%. (**b**) Pharmacokinetics of renadirsen sodium in *mdx* mice after its single subcutaneous injection at doses of 1, 3, and 10 mg/kg, analyzed using LC-MS/MS. Blood samples were collected 5, 15, and 30 min, and 1, 2, 4, 8, and 24 h after administration. Data in (**a**) and (**b**) are shown as means ± SD; *n* = 3.

To investigate the pharmacological activity from the perspective of in vivo behavior of the AO, we first analyzed the stability and pharmacokinetics of renadirsen sodium in mice. To determine the stability, renadirsen sodium was incubated with mouse blood, followed by monitoring of the mass peak corresponding specifically to the unchanged form of full-length renadirsen using an LC-MS/MS system. The ratio of the peak area of renadirsen to that of the IS remained stable in mouse blood for at least 8 h (Figure 3a). Consistent with previous studies [18,19,22], these results demonstrated that renadirsen was stable throughout the entire incubation period. Pharmacokinetic analysis of renadirsen sodium was performed in male *mdx* mice following subcutaneous injection of 1, 3, and 10 mg/kg doses. The blood samples were collected at different time points after injection, and we assessed renadirsen levels using LC-MS/MS analysis (Figure 3b; Supplemental Tables S1 and S2). The plasma concentrations of the free form of renadirsen sodium rapidly decreased below the lower limit of quantification (<20.0 ng/mL) and were not detected in any of the plasma samples collected at 72 h after administration. The time to maximum plasma concentration (T_max_) was determined to be 0.25 h for all doses tested, showing that renadirsen was immediately absorbed after subcutaneous administration. The absolute bioavailability value at 3 mg/kg was calculated to be 73.9%. Systemic exposure increased in a dose-dependent manner.

### 3.3. ^14^C-Labeled Renadirsen Exhibited Broad Tissue Distribution and Long-Term Retention in Tissues, including the Heart, Diaphragm, and Skeletal Muscles

**Figure 4:**
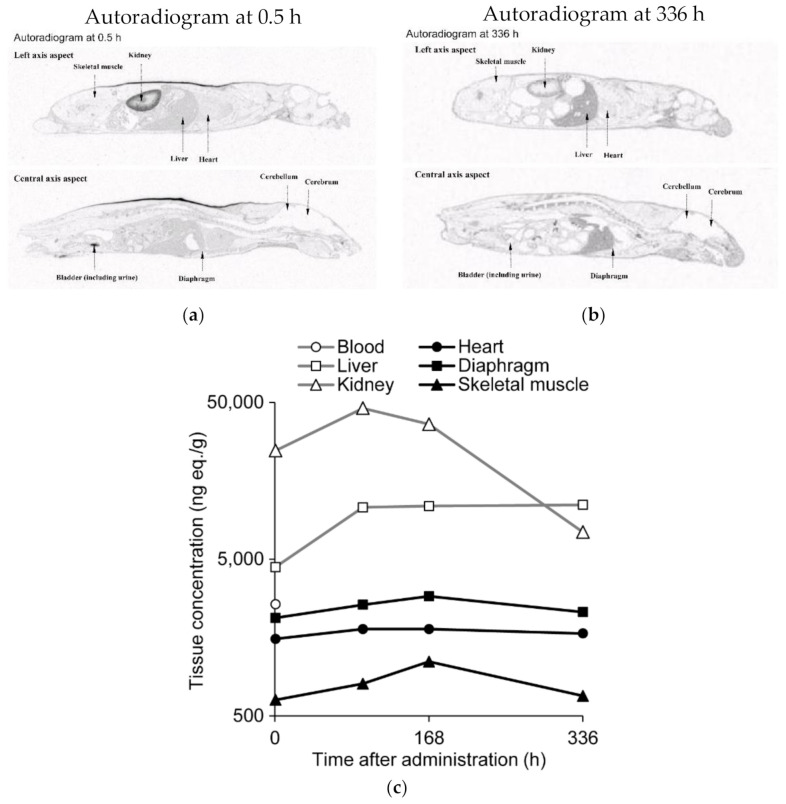
Concentrations of radioactivity in tissues after single subcutaneous administration of [^14^C] renadirsen sodium at 10 mg/kg in C57BL/6 mice. (**a**) Autoradiograms at 0.5 h after administration. (**b**) Autoradiograms at 336 h after administration. (**c**) Concentrations of radioactivity in tissues were quantified by whole-body autoradiography (QWBA) at 0.5, 96, 168, and 336 h after administration. *n* = 1 per time point. Radioactivity in the blood (open circle) decreased below the limit of quantification at 96 h after administration. Open squares, open triangles, closed circles, closed squares, and closed triangles represent the liver, kidney, heart, diaphragm, and skeletal muscle, respectively.

Next, to clarify the tissue distribution of renadirsen, a quantitative whole-body autoradiography (QWBA) study was performed after the single subcutaneous administration of 10 mg/kg [^14^C]renadirsen sodium to male C57BL/6J mice. At 0.5 h after administration, the radioactivity was extensively distributed in all tissues except for the cerebrum, cerebellum, spinal cord, and contents of the large intestine (Figure 4a). The highest radioactivity was observed in the kidney and the second highest in the bladder (including urine), indicating a potential elimination pathway via urine. At 96 h after administration, the radioactivity in the blood decreased below the detection limit, as with the previous pharmacokinetic analysis, while increased radioactivity was detected in the skeletal muscle, diaphragm, and heart, suggesting that the majority of the radioactivity was eliminated from the circulation via distribution to tissues (Figure 4c; Supplemental Table S3). Up to 336 h after administration, high levels of radioactivity were maintained in the heart and diaphragm, which were equal to or greater than the levels in the skeletal muscle (Figure 4b). Thus, the QWBA study demonstrated broad tissue distribution and long-term retention of renadirsen in muscles up to at least 336 h after administration. At each time point, higher levels of radioactivity were detected in the heart and diaphragm than in the skeletal muscle. These results indicate that renadirsen distributed in muscles may promote continuous exon skipping even after elimination from systemic circulation, and are consistent with efficient exon skipping observed 1 week after a single administration.

### 3.4. In Vitro Protein Binding of ^14^C-Labeled Renadirsen to Mouse Plasma

One of the most important factors characterizing the pharmacokinetics and tissue distribution profiles of AOs is the percentage of binding to plasma proteins. Tissue bioavailability is well known to be enhanced by plasma protein binding, which limits the glomerular filtration and ultimate urinary excretion of AOs [29]. To investigate the mechanism behind the long-term retention of renadirsen in tissues as shown in Figure 4c, the rate of in vitro protein binding of [^14^C]renadirsen sodium (1, 10, and 100 μg/mL) in mouse plasma (*n* = 3) was determined using an ultracentrifugation method. In the male mouse plasma samples spiked with [^14^C]renadirsen sodium, the plasma protein binding ratios were 96.5 ± 0.4% (mean ± SD), 97.4 ± 0.4%, and 97.7 ± 0.2% at 1, 10, and 100 μg/mL, respectively. No concentration-dependent change was noted over the range of concentrations tested. These results suggest that the high degree of plasma protein binding of renadirsen prevents renal filtration while enhancing the distribution of renadirsen in the studied tissues.

### 3.5. Renadirsen Showed Robust Exon-45-Skipping Activity in Skeletal and Cardiac Muscles Compared with 2′OMePS- and PMO-Based AO in mdx Mice

**Figure 5:**
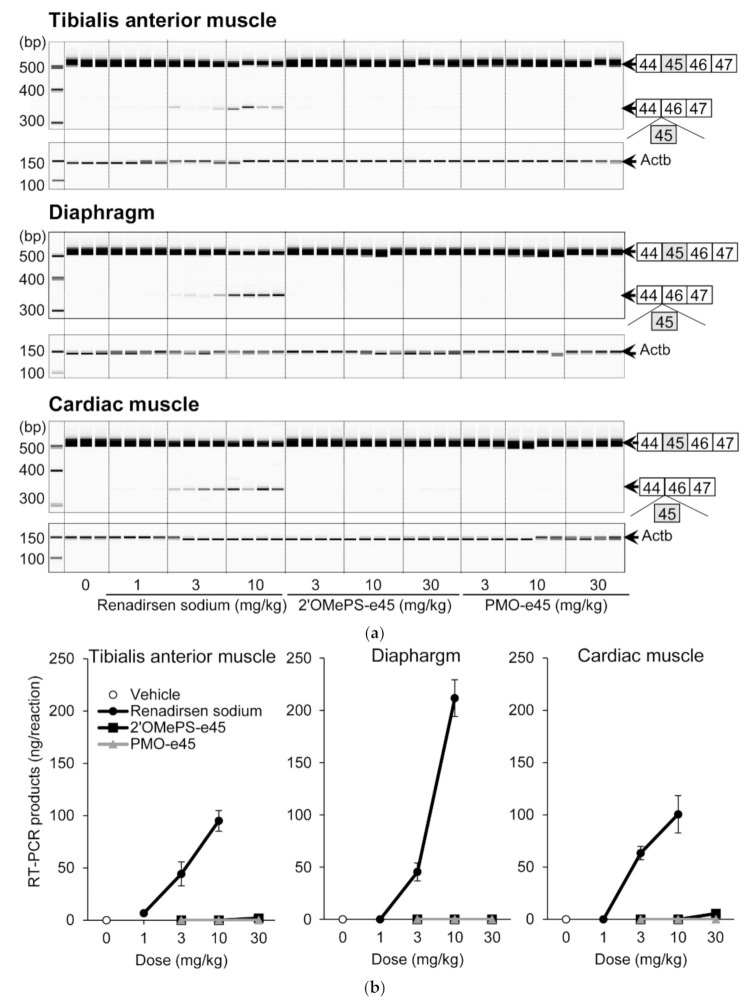
Effects of renadirsen sodium, 2′OMePS-e45, and PMO-e45 on dystrophin exon 45 skipping in *mdx* muscles. (**a**) Detection of exon-45-skipped RT-PCR products of dystrophin mRNA in various muscles from *mdx* mice after systemic delivery of renadirsen sodium, 2′OMePS-e45, or PMO-e45. Each compound was injected subcutaneously into *mdx* mice once a week for 4 weeks. Total RNA was isolated from the tibialis anterior muscle, diaphragm, and cardiac muscle. The structure of each PCR product is shown schematically at the right of the panel. (**b**) The concentrations of exon-45-skipped RT-PCR products of dystrophin mRNA from the tibialis anterior muscle, diaphragm, and cardiac muscle were calculated using an Agilent 2100 Bioanalyzer. Data in (B) are shown as means ± SEM; *n* = 3–4.

To compare the effectiveness of the 2′OMeRNA/ENA chimera with the phosphorothioate backbone to those of 2′OMePS and PMO, we performed a comparative pharmacology study in *mdx* mice repeatedly administered renadirsen sodium or AOs with the same sequence as renadirsen but conventionally modified with 2′-O-methyl RNA (2′OMePS-e45) or phosphorodiamidate morpholino oligomer (PMO-e45). These three AOs were injected subcutaneously into *mdx* mice once a week for 4 weeks, and their exon-skipping activities in the tibialis anterior muscle, diaphragm, and cardiac muscle were evaluated using RT-PCR (Figure 5a). Renadirsen sodium resulted in the skipping of exon 45 of mouse dystrophin in all samples taken from the tibialis anterior muscle, diaphragm, and cardiac muscle even at 3 mg/kg (Figure 5b). On the other hand, 2′OMePS-e45 achieved this skipping in one of four samples of tibialis anterior muscle and two of four samples of cardiac muscle at 30 mg/kg. Meanwhile, PMO-e45 did not show exon-skipping activity at any of the examined doses. These findings suggested that the chemistry of the 2′OMeRNA/ENA chimera on the phosphorothioate backbone made a major contribution to the robust exon-skipping activity in the various muscles, including cardiac muscle.

## 4. Discussion

In the present study, we showed the robust exon-skipping activity of renadirsen sodium in various muscles of *mdx* mice administered it subcutaneously at a dose of 3 mg/kg/week for 4 weeks. Notably, exon skipping was substantially promoted in the cardiac muscle, as well as in the tibialis anterior muscle and diaphragm. Furthermore, renadirsen sodium had significant activity at doses at which both 2′OMePS-e45 and PMO-e45 had little or no activity in *mdx* mice. 

To investigate the remarkable in vivo exon-skipping activity of renadirsen sodium, we analyzed several characteristics, including exon-skipping activity in muscles and stability in blood. For this, we performed pharmacokinetic analysis using LC-MS/MS and examined the tissue distribution via the QWBA study. The assay of the stability in blood demonstrated that renadirsen is highly stable against nucleases, which is consistent with previous studies of 2′OMeRNA/ENA [22]. The high stability of renadirsen was also confirmed by the fact that no metabolites were detected in mouse urine (data not shown), suggesting that the full-length molecule forms a major part of that distributed to tissues and excreted into urine. The PK study in *mdx* mice showed that renadirsen was immediately absorbed (T_max_ = 0.25 h) after subcutaneous administration, and the plasma concentrations decreased rapidly to below the lower limit of quantitation. In the QWBA study, renadirsen exhibited rapid distribution to tissues throughout the body, except for the central nervous system (CNS), and its high distribution in the kidney and urinary bladder containing urine suggested its urinary excretion. The high concentrations of renadirsen were observed in the kidney and liver, which can primarily be explained by the following three factors: (1) high blood flow that delivers more AOs to the tissues, (2) fenestrated or discontinuous capillaries where AOs easily leak into tissues, and (3) presence of phagocytically active cells that take up AOs. Although high levels of distribution of renadirsen were observed in the liver and kidney, GLP studies in mice showed no severe toxicological findings in those tissues. The transient high levels of renadirsen in the kidney after administration are presumably due to rapid glomerular filtration of unbound renadirsen and uptake into renal proximal tubular cells, and then the concentrations gradually decreased over time due to excretion into urine. The concentrations of renadirsen were found to be relatively high in endocrine tissues such as the thyroid because they also have fenestrated capillary. Phosphorothioated AOs are known to distribute in connective tissues including the submucosa of the intestine and the dermis and subcutaneous layer of the skin [30], which is consistent with the observations for renadirsen. Based on these findings, the rapid clearance of renadirsen from systemic circulation can be explained by its rapid distribution to tissues and urinary excretion in an unchanged form. The distribution and long-term retention of renadirsen in the target muscle tissues, such as skeletal muscle, diaphragm, and heart, are considered to contribute to its long-lasting exon-skipping activity, as demonstrated by the observation of exon-skipped dystrophin mRNA even 1 week after its subcutaneous administration. The concentrations of renadirsen in cardiac muscle were higher than those in skeletal muscle, and one of the possible explanations for the difference is that the blood flow per unit volume in cardiac muscle is higher than that in skeletal muscle. Similar profiles of stability and distribution were also observed for AOs modified with 2′OMePS, as reported in a previous study, where 2′OMePS-modified AOs targeting dystrophin exon 23 were rapidly eliminated from systemic circulation through urinary excretion and taken up into multiple tissues, not including the CNS [31]. In previous studies, the half-lives of the 2′OMePS-modified AOs were found to be 10 days in the gastrocnemius and quadriceps muscles, and markedly longer in the triceps muscle (~33 days) and in cardiac muscle tissue (~46 days). In the 14-day QWBA study, renadirsen was persistently detected in muscle tissues, and no clear decrease in radioactivity was observed over the study period, suggesting a long half-life of renadirsen comparable or superior to those of 2′OMePS-modified AOs. 

Although several similarities were found between 2′OMeRNA/ENA and 2′OMeRNA as discussed above, there were also some differences between them. Previous reports have demonstrated that the 2′OMeRNA/ENA chimera on the phosphorothioate backbone is superior to 2′OMePS with regard to nuclease resistance [18,19,32,33]. Additionally, from a thermodynamic perspective, ENA modification is more stable (DNA +3.5–5.2 °C, ΔTm/modification) than 2′OMe modification (DNA +0.2–1.4 °C, ΔTm/modification); furthermore, a higher affinity of ENA modification toward mRNA has also been reported [18,19]. The physicochemical properties of renadirsen sodium evidenced by these comparisons could explain the higher accumulation of renadirsen in the tissues after repeated administration compared with the findings for 2′OMePS-modified AOs. This may be one of the reasons why repeated administrations of renadirsen sodium showed more potent exon-skipping activity than those of 2′OMePS-e45 in the target tissues, including cardiac muscle, in the *mdx* mouse study. 

The difference in the exon-skipping activity between renadirsen sodium and the PMO-type drug was also notable in our study. We compared our results to the ADME profiles in mice of eteplirsen, an AO targeting exon 51 with a PMO backbone [34]. The AUC of plasma concentration and the distribution of renadirsen sodium at a dose of 10 mg/kg s.c. in the skeletal muscle, diaphragm, and cardiac muscle of *mdx* mice were comparable to those of eteplirsen at a dose of 120 mg/kg, i.v. The difference in the dose required to exert pharmacological effects could be explained by the plasma protein binding of AOs, which prevents elimination via renal filtration while facilitating tissue uptake. AOs on the phosphorothioate backbone extensively bind (≥85%) to plasma proteins [35]; conversely, plasma protein binding of PMOs is low (e.g., eteplirsen in mice: 17.9%–25.4%) [29,36]. The higher degree of protein binding of renadirsen appears to result in higher drug exposure to the target tissues, leading to more potent exon-skipping activity than that of PMO-e45. In addition, the difference in AO uptake into target tissues and cells could be another factor explaining the difference in exon-skipping activity between renadirsen and PMOs. Previously, unassisted AO uptake in normal myotube cells, known as “gymnosis,” was reported for AOs with a phosphorothioate backbone [37,38,39], but not for PMO, and PMO delivery into muscle required the accumulation and retention of PMO within inflammatory foci associated with dystrophic lesions, and the fusion of PMO-loaded myoblasts into repairing myofibers [40]. Therefore, PMO is not expected to be delivered in terminally differentiated or nonregenerative cells such as cardiomyocytes. Furthermore, in in vivo studies, the systemic administration of PMO in healthy WT mice was not as effective as that in *mdx* mice under the same dosing regimens [41,42], whereas the systemic administration of renadirsen sodium produced clear exon-skipping activity even in WT mice in our study. Therefore, renadirsen is assumed to have cellular uptake superior to that of PMOs in muscle tissues and cells even under normal conditions. The mechanism of PMO uptake mediated through a process of destruction/regeneration in skeletal muscle is unlikely to work in cardiac muscle because of the lack of regenerative ability, which may be one of the reasons why renadirsen sodium has more potent effects on cardiac muscle cells, and on normal myotubular cells, than PMOs. Recent clinical studies have suggested that earlier intervention with exon-skipping drugs before tissue remodeling is more effective [43]. The potent effect of renadirsen sodium on normal cells, including cardiac cells, may enhance the efficacy of the early intervention and lead to a more marked clinical benefit, such as the prevention of cardiac failure and skeletal muscle wasting in DMD patients.

Since *mdx* mice, which have a nonsense mutation in exon 23 of dystrophin, were evaluated for exon-skipping activity only via mRNA expression levels, further research is necessary to demonstrate the functional improvements achievable via AOs. To show restoration of the dystrophin protein in the diaphragm and cardiac muscle and functional improvement of the pathology in DMD, especially for the cardiorespiratory system in a preclinical study, as previously reported in *mdx* mice [44], the establishment of a new mouse model, such as a mouse with deletion of exon 44 of the dystrophin gene or a humanized dystrophin gene [45], is required.

In summary, the present study showed that renadirsen sodium has robust exon-skipping activity in various muscles, especially in cardiac muscle in vivo, probably due to its superior characteristics compared with conventionally modified AOs, namely, its binding affinity to the target RNA, half-life in tissues, drug exposure, and uptake into target cells. The unprecedented high exon-skipping activity caused by renadirsen sodium in cardiac muscle may provide a whole new treatment option for suppressing cardiac dysfunction in DMD patients. Although further assessments for gymnosis in vitro and functional improvement using new DMD model mice are necessary, renadirsen sodium may be a promising exon-skipping drug that could improve various symptoms of patients with DMD, especially in terms of cardiorespiratory function, more efficiently than ever before.

## Data Availability

The data that support the findings of this study are not publicly available. However, data are available from the authors upon reasonable request.

## Supplementary Materials

The following are available online at https://www.mdpi.com/article/10.3390/cimb43030090/s1, Supplemental Table S1. Mean plasma concentrations with SD values after single administration of renadirsen sodium to mdx mice Table S2. PK parameters for renadirsen sodium after subcutaneous administration to male *mdx* mice. Table S3: Radioactivity concentration of renadirsen sodium (ng eq./g) in the various tissues collected from fasted male C57BL/6J mice at each time point.
