# Supplementary material for: Renadirsen, a Novel 2′OMeRNA/ENA® Chimera Antisense Oligonucleotide, Induces Robust Exon 45 Skipping for Dystrophin In Vivo
Source: Curr Issues Mol Biol. 2021 Sep 25;43(3):1267–81. doi: 10.3390/cimb43030090 (PMC8928966; doi:10.3390/cimb43030090)
Supplement: Supplementary file 1 [file cimb-43-00090-s001.zip › cimb-1337756-supplementary.pdf]

current issues in molecular biology

MDPI

Table S1. Mean plasma concentrations of renadirsen sodium with SD values after single administration to *mdx* mice.

Dose (mg/kg)		Concentration (ng/mL)									
		pre	5 min	15 min	30 min	1 h	2 h	4 h	8 h	24 h	72 h
1	Mean	0	462	843	781	538	233	56.0	0	0	0
	SD	0	146	125	28	18	29	6.7	0	0	0
3	Mean	0	1710	2200	2130	1840	598	197	52.9	0	0
	SD	0	250	200	80	110	99	71	9.3	0	0
10	Mean	0	4320	8430	7540	5300	2570	605	132	21.1	0
	SD	0	280	820	210	1030	510	6	18	1.2	0

Table S2. PK parameters for renadirsen sodium after subcutaneous administration to male *mdx* mice.

Dose (mg/kg)	T_{max} (h)	C_{max} (ng/mL)	AUC_{last} (ng·h/mL)	AUC_{all} (ng·h/mL)
1	0.25	843	1340	1450
3	0.25	2200	4450	4870
10	0.25	8430	16300	16800

Pharmacokinetic parameters were calculated based on the mean plasma concentration data of three animals for each time point.

Table S3. Concentration of radioactivity of renadirsen sodium (ng eq./g) in various tissues collected from fasted male C57BL/6J mice at each time point.

	0.5 h	96 h	168 h	336 h
Blood	2580	BLQ	BLQ	BLQ
Cerebrum	BLQ	BLQ	BLQ	BLQ
Cerebellum	BLQ	BLQ	BLQ	BLQ
Pituitary	2290	2150	1800	2230
Spinal cord	BLQ	BLQ	BLQ	BLQ
Eyeball	574	1030	304	479
Harderian gland	2790	2160	2130	2840
Submandibular lymph node	2150	7790	4340	5570
Submaxillary gland	5680	7990	7510	6280
Thyroid	2670	6040	4540	3380
Thymus	800	1830	2250	932
Heart	1550	1790	1790	1680
Lung	1830	594	1180	736
Liver	4450	10700	10900	11100
Kidney	24600	45900	36300	7440
Adrenal	2610	2420	2140	1700
Spleen	4390	8700	11400	6440
Pancreas	2590	2620	2550	2440
Prostate	1530	2460	3100	2920
Testis	184	1100	941	1220
Epididymis	1240	2150	2510	2710
Skin	2340	3980	3860	3530
Skeletal muscle	633	803	1110	671
Bone (femur)	559	1900	1950	2110
Bone marrow (femur)	2140	8520	8820	8600

White adipose tissue	180	1450	1410	645
Brown adipose tissue	2700	1120	915	1150
Bladder (including urine)	13900	1770	3470	1680
Gallbladder (including bile)	761	477	N.A.	1060
Mesenteric lymph node	3760	2290	4110	4620
Stomach	4020	2650	2240	4310
Small intestine	4580	3530	2660	3220
Large intestine	2250	5500	2120	3800
Contents of stomach	1240	177	BLQ	BLQ
Contents of small intestine	1750	730	205	614
Contents of large intestine	N.A.	335	279	BLQ
Diaphragm	2110	2560	2900	2300

BLQ: below the lower limit of quantification (<132 ng eq./g). N.A.: not applicable.
